# Supplementary material for: Drug repositioning of polaprezinc for bone fracture healing
Source: Commun Biol. 2022 May 16;5:462. doi: 10.1038/s42003-022-03424-7 (PMC9110432; doi:10.1038/s42003-022-03424-7)
Supplement: Supplementary file 2 — Supplementary Information [file 42003_2022_3424_MOESM2_ESM.pdf]

Supplementary data

Drug Repositioning of Polaprezinc for Bone Fracture Healing

Eun Ae Ko[1†], Yoo Jung Park[1†], Dong Suk Yoon[1†], Kyoung-Mi Lee[1,2], Jihyun Kim[1], Sujin Jung[1,2], Jin Woo Lee[1,2,3], Kwang Hwan Park[1]

[1]Department of Orthopaedic Surgery, Yonsei University College of Medicine, Seoul 03722, South Korea.

[2]Severance Biomedical Science Institute, Yonsei University College of Medicine, Seoul 03722, South Korea.

[3]Brain Korea 21 PLUS Project for Medical Science, Yonsei University College of Medicine, Seoul 03722, South Korea.

[†] Eun Ae Ko, Yoo Jung Park, and Dong Suk Yoon contributed equally to the work.

Correspondence to Professor Kwang Hwan Park, Department of Orthopaedic Surgery, Yonsei University College of Medicine, Seoul 03722, South Korea; khpark@yuhs.ac (Tel: +82 2 2228 2185)

Supplementary figures and table

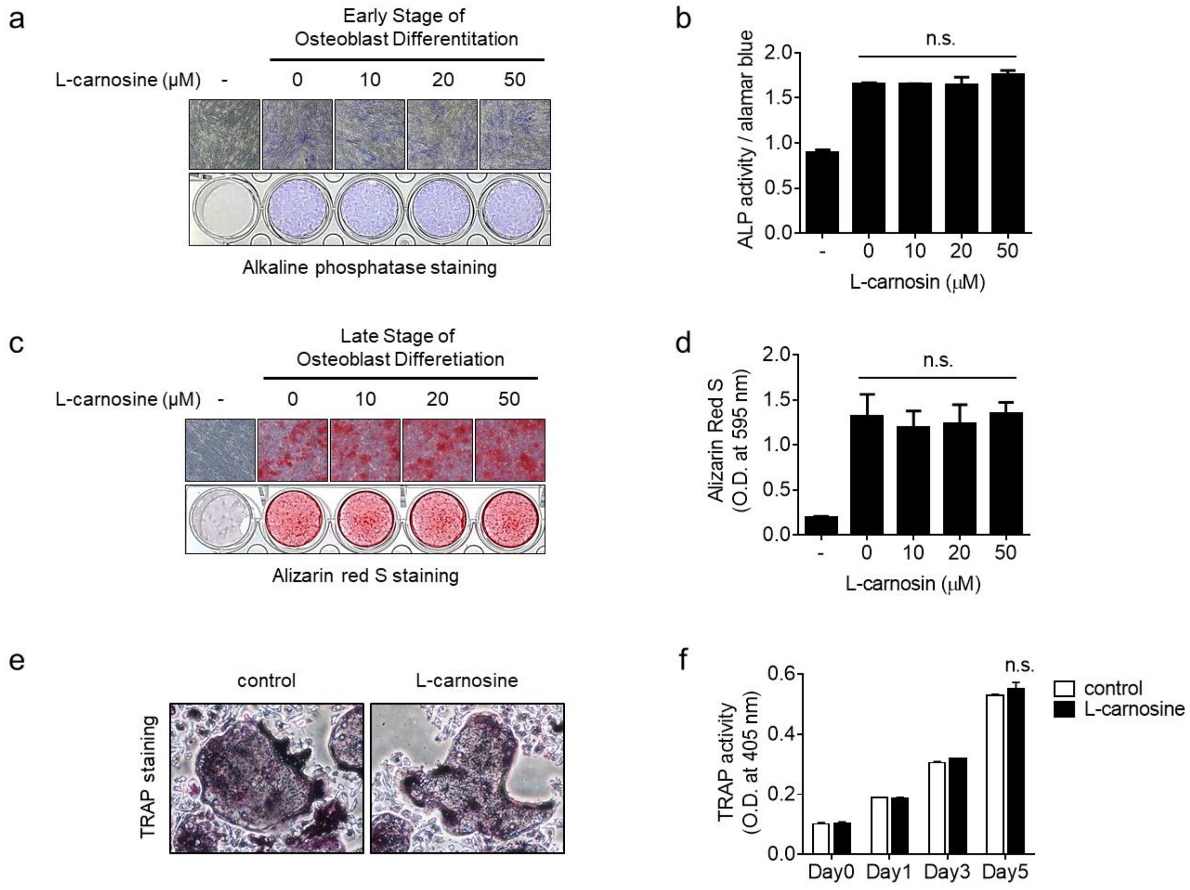

Supplementary figure 1. L-carnosine does not affect osteoblast and osteoclast differentiation. (a) Mesenchymal stem cells treated with vehicle or L-carnosine (10, 20, and 50 μM) were incubated in osteogenic medium for seven days. ALP staining was performed to determine the extent of the initial differentiation at day seven. (b) ALP activity assay was performed for the quantitative analysis of ALP staining. The absorbance was measured at 420 nm and normalized to that of alamar blue staining. (c) Alizarin red S staining was performed to detect mineral deposition at day 14. (d) For quantitative analysis of alizarin red S staining, absorbance was measured at 595 nm following destaining with 10% cetylpyridinium for 30 min.

(e) Representative images of osteoclast differentiation. Mouse BMMs treated with vehicle or L-carnosine were seeded in 12-well culture plates and treated for five days with osteoclastogenesis-related reagents (See materials and methods section). TRAP staining was performed to visulaize TRAP-positive BMMs. (f) TRAP activity was determined as described in the "materials and methods" section. The absorbance was measured at 405 nm and data are expressed as the mean ± S.D. (n = 3) from each independent experiment.

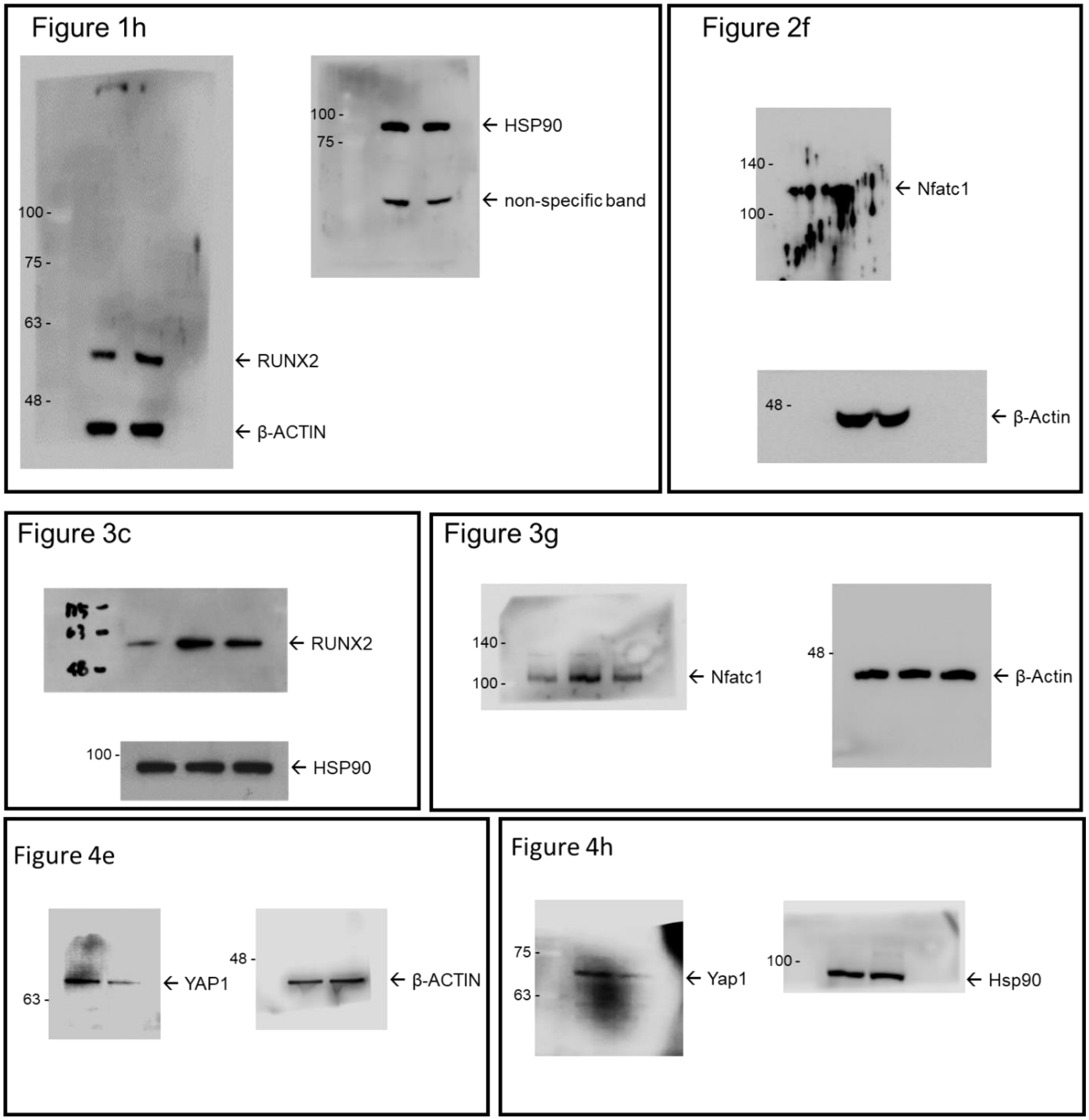

Supplementary figure 2. Uncropped blots of western blots performed in this study.

	PBS								p-zinc								*p-value*
	#1	#2	#3	#4	#5 (Excluded)	#6	#7	#8 (Excluded)	#1	#2 (Excluded)	#3	#4 (Excluded)	#5	#6	#7	#8	
Callus Volume (mm^3)	14.324	13.680	3.835	8.570	16.579	13.305	10.743	3.785	4.969	2.238	6.757	11.259	2.841	3.968	9.556	5.618	0.0069
BMD (g/cm^3)	0.576	0.523	0.545	0.626	0.458	0.587	0.571	0.708	0.623	0.456	0.653	0.781	0.676	0.737	0.687	0.685	0.0004
Callus Area (mm^2)	27.457	24.405	20.284	21.789	30.098	24.173	18.709	13.187	13.013	28.349	20.160	12.842	13.877	12.927	14.597	17.960	0.0123
Cartilage Area (mm^2)	18.564	11.895	5.461	6.454	18.878	7.519	5.530	2.837	2.016	0.825	1.713	3.352	2.754	3.040	1.971	0.847	0.0208
Bone Area (mm^2)	7.676	6.460	16.354	9.496	3.587	3.791	8.755	19.654	12.655	8.783	14.955	20.678	18.195	13.692	14.023	14.704	0.0266
Fibrotic Tissue Area (mm^2)	0.845	0.401	0.626	0.489	3.173	1.730	0.818	0.254	0.292	0.219	0.163	0.915	0.296	0.220	0.791	0.352	0.0072
Number of $TRAP^+$ cells / Total Area (mm^2)	22.2	17.1	37.7	23.6	8.6	28.5	31.8	41.3	39.5	12.2	39.5	88	80	36.7	44.4	48.8	0.0045

Supplementary Table 1. Raw data for histology data. This table is the full set of fracture data for the graphs shown in Figure 5.Groups excluded from statistical analysis are marked in red. Results were obtained from a total of 8 experimental animals, and the groups showing the highest and lowest values in each group were excluded from the analysis. Results with $p<0.05$ are statistically significant in the graphs of Figure 5.
