# Supplementary material for: Drug repositioning of polaprezinc for bone fracture healing
Source: Commun Biol. 2022 May 16;5:462. doi: 10.1038/s42003-022-03424-7 (PMC9110432; doi:10.1038/s42003-022-03424-7)
Supplement: Supplementary file 3 — Description of Additional Supplementary Files [file 42003_2022_3424_MOESM3_ESM.pdf]

Description of Additional Supplementary Files

File name: Supplementary Data 1
Description: Source data of the main and supplementary figures.
